# Association between COVID-19 and the incidence of type 1 diabetes in Portugal – a registry study

**DOI:** 10.1186/s12902-024-01667-5

**Published:** 2024-08-09

**Authors:** Morten Bjerregaard-Andersen, Jessica Da Silva, Rui Diogo, Ana Raquel Claro, Inês Ferro, Andreia Romana, Patrícia Rocha, Beatriz Sá, Goreti Lobarinhas, Sara Rolim, Claus Bogh Juhl, Kurt Højlund, Isabel Fernandes, Sónia Antunes, Maria Manuela Félix Calha, Guida Gama, Sofia Amálio, Mariana Figueiras, Teresa Silva, Margarida Rosado, Estela Ferrão, Luísa Arez, Ana Baptista, Adriana Martins Ferreira, Diana Alba, Carlos Godinho, Ana Luísa Leite, Maria de Lurdes Afonso Lopes, Maria Lurdes Sampaio, Joana Serra-Caetano, Eugenia Carvalho

**Affiliations:** 1 Department of Endocrinology and Nephrology, University Hospital of Southern Denmark, Finsensgade 35, 6700 Esbjerg, Denmark; 2 Steno Diabetes Center Odense, Odense University Hospital, Odense, Denmark; 3 Department of Regional Health Research, University of Southern Denmark, Odense, Denmark; 4 Institute for Interdisciplinary Research, Doctoral Program in Experimental Biology and Biomedicine (PDBEB), University of Coimbra, Coimbra, Portugal; 5 CNC-UC – Center for Neuroscience and Cell Biology, University of Coimbra, Coimbra, 3004-504 Portugal; 6 CIBB – Centre for Innovative Biomedicine and Biotechnology, University of Coimbra, Coimbra, 3004-504 Portugal; 7 Hospital Pediátrico de Coimbra, Centro Hospitalar e Universitário de Coimbra (CHUC) E.P.E., Coimbra, Portugal; 8 Departamento de Pediatria, Hospital de Santa Maria, Centro Hospitalar Universitário Lisboa Norte (CHLN) E.P.E., Lisbon, Portugal; 9 Centro Hospitalar de Leiria E.P.E., Leiria, Portugal; 10 Hospital Santa Maria Maior E.P.E., Barcelos, Portugal; 11 Hospital Espírito Santo E.P.E., Evora, Portugal; 12 Centro Hospitalar Universitário do Algarve (CHUA) E.P.E., Faro, Portugal; 13 Centro Hospitalar do Tâmega e Sousa E.P.E., Guilhufe, Portugal; 14 Centro Hospitalar de Vila Nova de Gaia/Espinho (CHVNG/E) E.P.E., Vila Nova de Gaia, Portugal; 15 Unidade de Endocrinologia Pediátrica, Hospital de Dona Estefânia, Centro Hospitalar Universitário de Lisboa Central (CHULC) E.P.E., Lisbon, Portugal; 16 Institute for Interdisciplinary Research (IIIUC), University of Coimbra, Casa Costa Alemão, Coimbra, 3030- 789 Portugal

**Keywords:** Type 1 diabetes, COVID-19, Portugal

## Abstract

**Background:**

Viral respiratory infections may precipitate type 1 diabetes (T1D). A possible association between the severe acute respiratory syndrome coronavirus 2 (SARS-CoV-2), the virus responsible for COVID-19, and the incidence of T1D is being determined. This study was carried out using Portuguese registries, aiming at examining temporal trends between COVID-19 and T1D.

**Methods:**

Hospital data, comparing the incidence before and during the COVID-19 pandemic, from children and young adults diagnosed with new-onset T1D, was acquired beginning in 2017 and until the end of 2022. Data was obtained from nine different Portuguese hospital units. The impact of the COVID-19 pandemic, beginning in March 2020, was assessed comparing the annual numbers of new-onset T1D cases. The annual median levels of glucose, glycated hemoglobin (HbA1c) and fasting C-peptide at T1D diagnosis were compared. The annual number of diabetic ketoacidosis (DKA) episodes among new T1D cases was also assessed at two centers.

**Results:**

In total, data from 574 newly diagnosed T1D patients was analyzed, including 530 (92.3%) children. The mean ages for child and adult patients were 9.1 (SD 4.4) and 32.8 (SD 13.6) years, respectively. 57.8% (331/573) were male, one patient had unknown sex. The overall median (25–75 percentiles) levels of glucose, HbA1c and fasting C-peptide at diagnosis were 454 mg/dL (356–568), 11.8% (10.1–13.4) and 0.50 µg/L (0.30–0.79), respectively. DKA at T1D diagnosis was present in 48.4% (76/157). For eight centers with complete 2018 to 2021 data (all calendar months), no overall significant increase in T1D cases was observed during the COVID-19 pandemic, i.e. 90 cases in 2018, 90 cases in 2019, 112 in 2020 and 100 in 2021 (P for trend = 0.36). Two of the centers, Faro (CHUA) and Dona Estefânia (CHULC) hospitals, did however see an increase in T1D from 2019 to 2020. No significant changes in glucose (*P* = 0.32), HbA1c (*P* = 0.68), fasting C-peptide (*P* = 0.20) or DKA frequency (*P* = 0.68) at the time of T1D diagnosis were observed over the entire study period.

**Conclusion:**

The T1D incidence did not increase significantly, when comparing the years before and during the COVID-19 pandemic, nor did key metabolic parameters or number of DKA episodes change.

## Background

Type 1 diabetes (T1D) belongs to the group of autoimmune diseases and is characterized by gradual destruction of the pancreatic ß-cells, resulting in insulin deficiency. Although the clinical manifestations of T1D are well characterized, with autoimmune biomarkers found in 70–90% of the patients [1], the exact pathogenesis remains elusive. It has been hypothesized that certain viral infections, including those caused by enterovirus, are involved and can induce T1D [2, 3]. The proposed mechanisms include both direct pancreatic damage and the precipitation of autoimmune development [4]. In Northern Europe, the T1D incidence is usually highest during the winter, where respiratory viral infections are also most common, providing further support for the virus induced T1D theory [5, 6].

The severe acute respiratory syndrome coronavirus 2 (SARS-CoV-2) is the virus linked to the coronavirus disease 2019 (COVID-19), which became a pandemic during March 2020, affecting virtually all countries [7]. Though COVID-19 symptoms are predominantly respiratory, the SARS-CoV-2 can infect many parts of the body, through binding to the angiotensin-converting enzyme 2 (ACE-2) receptor, which is also densely distributed in the pancreas [8, 9]. Early during the COVID-19 pandemic it was hypothesized that the SARS-CoV-2 could induce T1D. Thus, a marked increase in T1D cases was found at a pediatric center in the United Kingdom from March to June 2020 [10]. Since then, a number of mainly register based studies have investigated this association and the results have been conflicting. A large investigation from the United States, based on the medical insurance claims databases, found a higher incidence of diabetes among young patients < 18 years after SARS-CoV-2 infection [11], while a study from Scotland with detailed information on the date of T1D diagnosis and SARS-CoV-2 PCR testing did not replicate this finding [12]. A meta-analysis including 26 papers reported an increase in T1D [13], as did another recently published systematic review of 42 studies with meta-analysis [14], though data from the worldwide SWEET register did not find evidence of a link between COVID-19 and T1D [15]. A large Canadian cohort study recently reported an increase in the diabetes frequency following the COVID-19 pandemic, but it could not establish a correlation specifically for T1D, possibly due to the low T1D numbers [16]. In Denmark, no association was observed in a prospective register investigation among children and young adults [17], and another Danish register study evaluating the entire population below 30 years similarly reported no association [18].

In the present multicenter study, we report incidence data on new-onset T1D cases across Portugal before and during the COVID-19 epidemic to determine its impact on T1D development. We further provide an analysis on the changes in glycemic levels and endogenous insulin production at T1D diagnosis, before and during the epidemic. Finally, we evaluate the annual number of diabetes ketoacidosis (DKA) episodes, as well as previous COVID-19 infection as a risk factor for DKA at the time of T1D diagnosis.

## Methods

### Data collection

The study was a retrospective and hospital-based evaluation comprising new-onset T1D cases and COVID-19 related data from nine hospital units across Portugal, from January 2017 to December 2022. Complete data for all calendar months were, however, only available from eight centers from 2018 to 2021.

Most sites were pediatric centers, including the hospitals Santa Maria and Dona Estefânia in Lisbon, Centro Hospitalar in Leiria, Hospital Santa Maria Maior in Barcelos, Hospital Espírito Santo in Évora, Centro Hospitalar Universitário do Algarve in Faro, the Pediatric Hospital in Coimbra, and the pediatric unit at Centro Hospitalar de Vila Nova de Gaia/Espinho. Only one adult center, Hospital de Portimão e Lagos at the Algarve Coast, participated.

The Centro Hospitalar de Vila Nova de Gaia/Espinho did not have complete data available from the last part of 2021 and was therefore not included in the T1D case trend analysis. It was, however, included in the analysis of the metabolic parameters.

### Data variables

The demographic and clinical information of new-onset T1D cases included age, gender, symptoms at diabetes diagnosis, height, weight, blood pressure, family history of T1D (any relative), other illnesses and COVID-19 infection (usually PCR or Quick-test confirmed) prior to or concurrent with T1D diagnosis.

The Bacillus Calmette-Guérin (BCG) vaccine has previously been suggested as a possible protective factor in terms of COVID-19 severity [19], and we therefore recorded previous BCG vaccination. For one center, data could also be extracted on prior COVID-19 vaccination.

Biochemical data included initial random glucose and glycated hemoglobin (HbA1c) at first presentation (time of diagnosis), fasting C-peptide (as a measure of endogenous insulin production), low density lipoprotein (LDL), total cholesterol and the presence of anti-glutamic acid decarboxylase (GAD) and Islet antigen-2 (IA-2) antibodies. Total cholesterol was considered high if above 200 mg/dL, LDL if above 130 mg/dL. Data about initial presentation with diabetes ketoacidosis (DKA) was available for only two centers.

### Statistical analysis

Demographic, clinical, vaccination and biochemical characteristics of the participants were summarized in percentages for categorical variables. For continuous variables, the data were reported in means (with standard deviation (SD)) if normally distributed and in medians (25–75 percentiles) if not normally distributed.

The impact of the COVID-19 epidemic was assessed by comparing the annual numbers of new-onset T1D cases, using linear regression for trend analysis, from 2018 to 2021. Median levels of glucose, HbA1c and fasting C-peptide at T1D diagnosis were analyzed using Kruskal-Wallis test, due to the non-normal distribution of the data. In these metabolic analyses, we used the entire dataset, from 2017 to 2022.

The annual incidence of DKA cases was assessed using linear regression. The risk factor assessment for DKA at time of T1D diagnosis, with prior COVID-19 as the main variable of interest, was done using logistical regression and reported as odds ratios (OR). Concurrent COVID-19 identified at the time of T1D diagnosis was included in the prior COVID-19 group, as test results can remain positive for some time. In the multivariate model, we included co-variates previously investigated in relation to DKA such as age, gender, family history of T1D and HbA1c [20, 21]. Age was included as a continuous variable. HbA1c was grouped (≤ 12.0, > 12.0%) due to non-linearity, with the 12.0% cut-off chosen for being close to the overall HbA1c median and the fact that values above that indicate severe glycemic dysregulation.

All data were analyzed in STATA 15 software (StataCorp LLC, Texas, USA).

## Results

### Demographic characteristics

A total of 574 patients with new-onset T1D were included in this analysis. Due to the centers included, the vast majority were children below 18 years of age, i.e. 92.3%. (530/574). The mean ages for child and adult T1D cases were 9.1 (SD 4.4) and 32.8 (SD 13.6) years, respectively. Slightly more males than females were included, i.e. 57.8% (331/573). The sex of one patient was not available. A family history of T1D was reported in 20.9% (113/541) of the participants. A total of 8.6% (42/487) reported prior (or concurrent) COVID-19 infection at T1D diagnosis. The majority, 81.1% (413/509), had previously been BCG vaccinated. The results are reported in Table 1.

**Table 1:** Patients in Portugal presenting with newly diagnosed type 1 diabetes

	Hospital Espírito Santo *N* = 43	Centro Hospitalar de Leiria *N* = 34	Hospital Santa Maria Maior *N* = 21	Hospital de Dona Estefânia (CHULC) *N* = 60	Hospital de Santa Maria *N* = 107	Hospital de Faro (CHUA) *N* = 78	Hospital de Portimão e Lagos (CHUA) *N* = 50	Centro Hospitalar de Coimbra (CHUC) *N* = 127	Centro Hospitalar de Vila Nova de Gaia/Espinho (CHVNG/E) *N* = 54
**Demographic and clinical data**									
Age (years)^1^	8.58 (4.71)	10.3 (4.97)	11.2 (3.17)	7.97 (4.02)	9.06 (4.52)	8.96 (4.22)	32.8 (13.6)	9.29 (4.48)	8.78 (4.12)
Male sex (%, n/N)	56% (24/43)	53% (18/34)	52% (11/21)	53% (32/60)	64% (68/106)	46% (36/78)	66% (33/50)	63.0% (80/127)	53.7% (29/54)
Weight (kg)^1^	31.7 (15.8)	37.3 (18.3)	38.9 (10.2)	29.1 (12.7)	34.0 (17.8)	34.8 (16.9)	63.2 (15.4)	37.0 (18.6)	36.2 (17.8)
Height (cm)^1^	131.4 (29.2)	148.4 (27.1)	150.1 (15.5)	131.4 (25.2)	135.9 (28.6)	136.6 (27.3)	167.9 (10.2)	138 (27.6)	136.7 (25.8)
Systolic BP (mmHg)^1^	111.1 (13.4)	114.8 (12.2)	112.2 (10.3)	106.0 (19.5)	110.3 (11.6)	109 (12.8)	113 (14.5)	104.6 (12.9)	105.1 (12.1)
Diastolic BP (mmHg)^1^	69.8 (11.3)	70.4 (10.7)	64.9 (8.4)	66.8 (9.8)	64.4 (10.3)	68.4 (10.7)	72.5 (12.2)	60.8 (9.53)	57.8 (6.35)
Family history of T1D (%, n/N)	28% (12/43)	32% (11/34)	-	34% (18/53)	22.4% (24/107)	19% (15/78)	16% (8/50)	11.0% (14/127)	21% (11/52)
Other comorbidities (%, n/N)	28% (12/43)	-	29% (6/21)	26% (15/58)	10.3% (11/107)	12% (9/78)	40% (19/48)	29.1% (37/127)	22% (11/51)
COVID-19 prior to T1D (%, n/N)	-	-	5% (1/21)	4% (2/53)	8.5% (9/106)	13% (10/78)	8% (4/50)	11.8% (15/127)	2% (1/52)
**Vaccination status**									
COVID-19 vaccination (%, n/N)	-	-	-	-	-	-	18% (9/50)	-	-
BCG vaccination (%, n/N)	72.1% (31/43)	88.2% (30/34)	95.2% (20/21)	78% (47/60)	84.8% (89/105)	80.8% (63/78)	58.5% (24/41)	85.8% (109/127)	-
**Biochemical data**									
Glucose (mg/dl)^2^	449 (388–515)	431 (361–663)	523 (421–556)	455 (359–539)	458 (370–600)	398 (310–498)	343 (254–500)	483 (381–595)	482 (375–580)
HbA1c (%), ^2^	11.6 (10.2–13.4)	11.8 (10.1–13.2)	10.4 (9.3–11.7)	11.7 (10.4–13.4)	12.3 (11.0-14.2)	11.3 (9.2–13.2)	12.2 (10.3–14.0)	11.9 (10.3–13.4)	11.6 (9.4–13.6)
Fasting C-peptide (µg/L)^2^	0.60 (0.35-1.0)	0.50 (0.30–0.75)	0.36 (0.23–0.59)	0.30 (0.20–0.40)	0.57 (0.40–0.87)	0.50 (0.30–0.90)	0.50 (0.20–0.70)	0.49 (0.30–0.70)	0.63 (0.52–0.96)
LDL (mg/dL)^2^	85 (68–127)	111 (83–163)	87.5 (79–102)	111 (92–147)	74 (61–91)	98 (78–116)	112 (85–140)	100 (81–125)	-
Total cholesterol (mg/dL)^2^	182 (140–211)	198 (168–264)	172 (155–203)	177 (153–201)	151 (135–162)	164 (143–189)	178 (149–212)	169 (147–210)	-
Presenting with DKA (%, n/N)	-	-	-	-	55.1% (59/107)	-	34% (17/50)	-	-
GAD antibody positive (%, n/N)	61% (25/41)	75% (21/28)	86% (18/21)	65% (36/55)	96.0% (97/101)	88% (58/66)	62% (26/42)	88.9% (104/117)	-
IA-2 antibody positive (%, n/N)	63% (22/35)	-	89% (8/9)	67% (34/51)	75.0% (75/100)	80% (35/44)	54% (7/13)	80% (40/50)	100% (1/1)

^1^ Mean values (SD)

^2^ Median values (25-75 percentiles)

*Note* For abbreviations, CHULC denotes Centro Hospitalar Universitário de Lisboa Central, CHUA Centro Hospitalar Universitário Do Algarve, CHUC Centro Hospitalar e Universitário de Coimbra, CHVNG/E Centro Hospitalar de Vila Nova de Gaia/Espinho; BP Blood pressure, T1D Type 1 diabetes, COVID-19 coronavirus disease 2019, BCG Bacillus Calmette-Guérin, HbA1c glycated haemoglobin, LDL low density lipoprotein, DKA diabetes ketoacidosis, GAD glutamic acid decarboxylase, IA-2 islet antigen 2.

### Number of new-onset T1D cases

**Fig. 1:**
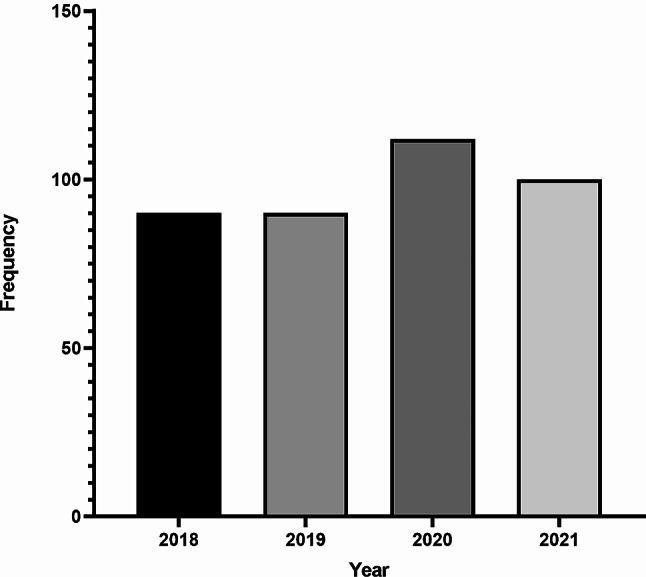
Number of newly diagnosed T1D patients per year (2018–2021). *Note* The figure only shows 2018-2021, as these were full calendar years. Also, it only includes the eight centers with complete data (i.e. Santa Maria Hospital in Lisbon, Dona Estefânia Hospital in Lisbon, Centro Hospitalar in Leiria, Hospital Santa Maria Maior in Barcelos, Hospital Espírito Santo in Évora, Centro Hospitalar Universitário do Algarve in Faro, the Pediatric Hospital in Coimbra, Hospital de Portimão e Lagos at the Algarve Coast).

Overall, in the eight centers with complete data (2018–2021), we observed a numerical increase in new-onset T1D cases during the study years. However, this did not reach statistical significance, i.e., 90 cases in 2018, 90 cases in 2019, 112 in 2020 and 100 in 2021 (P for trend = 0.36). The increase was largely driven by two sites, Faro (CHUA) and Dona Estefânia (CHULC) hospitals, which observed 2.6–2.8 times as many T1D cases in 2020 compared to 2019. The other hospitals reported unchanged annual T1D rates, or even lower in 2020. The annual T1D numbers from 2018 to 2021 for the eight centers combined are illustrated in Fig. 1.

**Fig. 2:**
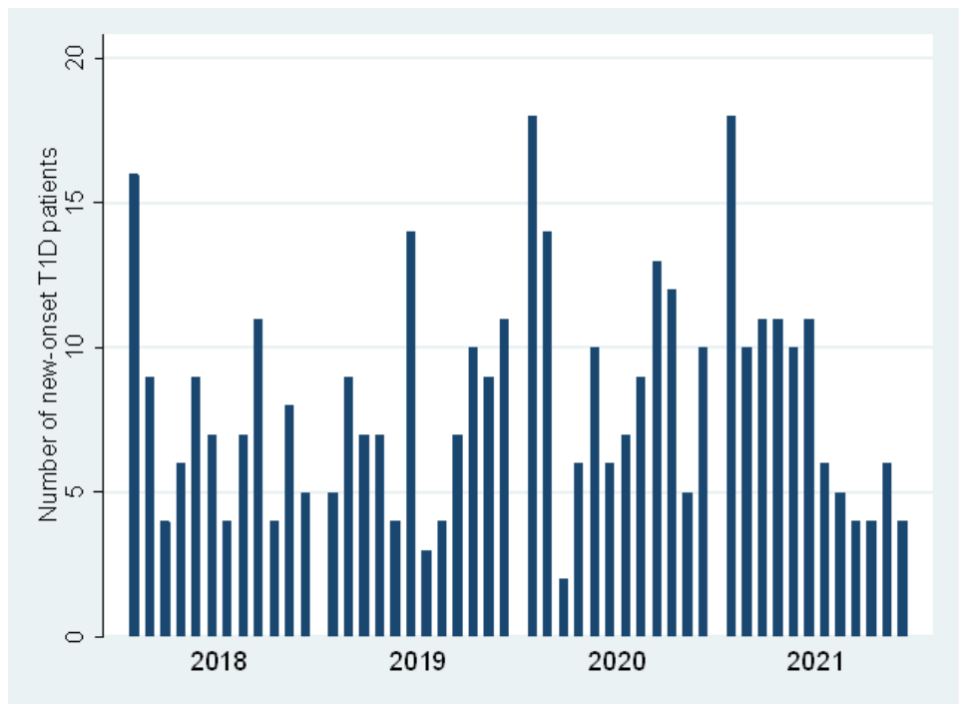
Number of newly diagnosed T1D patients per individual calendar month (2018–2021)

In terms of individual calendar months, the highest T1D case numbers were observed in January 2020 and January 2021 (18 cases in each month). The lowest rate was in March 2020, with only two new-onset T1D cases. The number of new-onset T1D cases per individual month from 2018 to 2021 is illustrated in Fig. 2.

The Centro Hospitalar de Vila Nova de Gaia/Espinho was not included in the case trend analysis, nor in Figs. 1 and 2, due to incomplete data from 2021. The numbers of new T1D cases at the center were 12 (2018), 17 (2019), 8 (2020), i.e. no increase was observed during the first year of the pandemic.

### Metabolic parameters

**Fig. 3:**
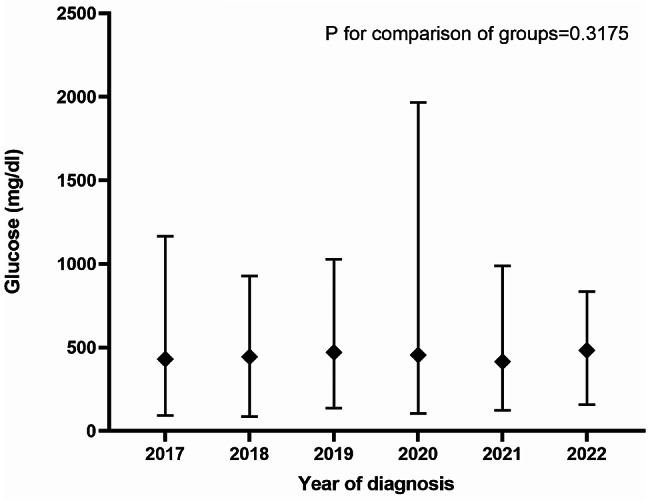
Glucose levels at T1D diagnosis from 2017 to 2022

**Fig. 4:**
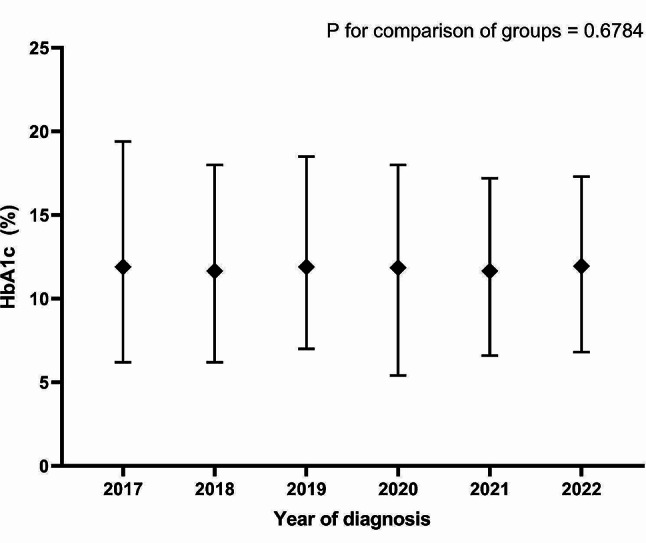
HbA1c levels at T1D diagnosis from 2017 to 2022

**Fig. 5:**
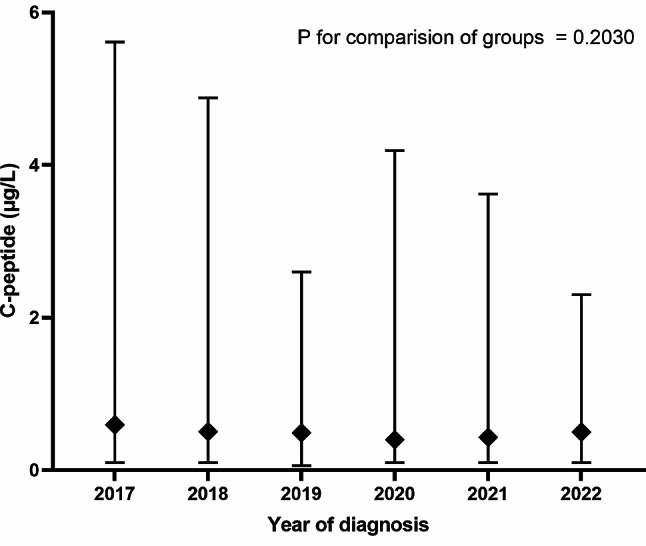
Fasting C-peptide levels at T1D diagnosis 2017 to 2022

The glucose, HbA1c and fasting C-peptide values at the time of T1D diagnosis from the individual hospital units are shown in Table 1, and the trend over time is illustrated in Figs. 3, 4 and 5. Overall, the median values were 454 mg/dL (356–568) for glucose, 11.8% (10.1–13.4) for HbA1c and 0.50 µg/L (0.30–0.79) for fasting C-peptide. No significant changes in the three biochemical analyses were observed during the entire study period from 2017 to 2022, i.e. *P* = 0.32, *P* = 0.68 and *P* = 0.20, respectively.

Overall, 81.7% (385/471) of the patients were GAD-antibody positive. This proportion tended to increase during the COVID-19 pandemic, compared to the time before, i.e. 85.6% (178/208) vs. 78.7% (207/263) (*P* = 0.06). For IA-2 antibodies, a total of 73.3% (222/303) were positive. This proportion did not increase during the pandemic, compared to the years before, 70.0% (105/150) vs. 76.5% (117/153) (*P* = 0.20).

The proportion of T1D patients with elevated total cholesterol and LDL levels did not increase significantly during the pandemic, compared to the years before. For cholesterol, the proportion was 27.6% (50/181) vs. 21.7% (55/254) (*P* = 0.15). For LDL, it was 20.8% (36/173) vs. 16.9% (42/248) (*P* = 0.31).

### DKA episodes and risk factor analysis

DKA data were available from a subset of 157 patients from two centers, i.e. children from Hospital Santa Maria in Lisbon and adults from Hospital de Portimão e Lagos (CHUA). The overall proportion of DKA episodes out of the total number of new T1D cases was 48.4% (76/157). The annual DKA incidence did not change during the full calendar years from 2018 to 2021 (*P* = 0.68).

The DKA risk factor analysis is shown in Table 2 and comprised all 157 patients with available DKA data (155 in the multivariate analysis). In both the univariate (*P* = 0.68) and multivariate model (*P* = 0.65), we could not establish prior COVID-19 infection as a risk factor for DKA. In the multivariate model, the OR for DKA decreased with higher age (0.97, 0.95-1.00), meaning that young age was a risk factor for DKA. There was a tendency of family history of T1D being a protective factor for DKA (0.52, 0.23–1.17).

## Discussion

### Main findings

Though a slight numerical increase was observed in new-onset T1D in 2020 and 2021, compared to the previous years, this did not reach statistical significance. Nor did glucose, HbA1c or fasting C-peptide levels and incidence of DKA at T1D diagnosis change significantly over the study years. As such, a strong association between COVID-19 and T1D was not confirmed.

### Strengths and limitations

Our study is the first national investigation of the relationship between new-onset T1D and COVID-19 in Portugal. It comprises a large dataset with considerable geographic variation and a T1D diagnosis confirmed at the individual level. In this context, the presence of GAD antibodies in 82% of the participants equals what has previously been reported for T1D patients [1], as does any family history of T1D in one fifth of the patients [22]. Importantly, apart from T1D incidence data, it also provides information on initial glycemic levels and fasting C-peptide across the years, as well as the number of and risk factors for presentation with DKA.

Our study does, however, have some limitations. Notably, we did not have access to SARS-CoV-2 antibody measurements. Furthermore, a detailed COVID-19 test history (including positive PCR or Quick-test result) may not always have been confirmed at the time of T1D diagnosis, and the 8.6% of patients with previous or concurrent COVID-19 could therefore be an underestimation. This naturally affects our possibility to conduct correlation analyses on individual patient level.

Our analysis only comprises data from the initial presentation, including fasting C-peptide. Hypothetically, infection with the SARS-CoV-2 virus may affect the progression of T1D, inducing a more rapid loss of endogenous insulin production through ß-cell apoptosis, but prospective longitudinal studies are necessary to test this hypothesis.

Our investigation of new T1D cases only includes absolute numbers from the individual hospitals, i.e. not relative to the background population. T1D incidence rates have therefore not been reported. Changes in patient uptake areas could have affected the numbers. However, we have no knowledge of any major changes in procedures here.

While symptoms at diagnosis were recorded for all centers, data on whether there was ketoacidosis could only be retrieved from a subset of two hospitals, which limits the precision and power of this analysis.

Finally, our data is largely based on pediatric T1D patients. We can therefore only make very limited assumptions in terms of adults with new-onset T1D. However, the highest incidence of T1D is usually seen before the age of 18 years [23].

### Consistency with previous findings

Portugal recorded the first cases of COVID-19 in early March 2020. As in other European countries, and despite strict control measures, a rapid increase in COVID-19 cases were observed over the following weeks [24]. Several severe COVID-19 waves were observed, and a population-based study reported a high SARS-CoV-2 seroprevalence of 17.3% in March 2021 [25].

While we did not observe an overall significant increase in the number of T1D cases during the COVID-19 pandemic, we did note a substantial rise at two individual centers. One was the Dona Estefânia Hospital in Lisbon, where an investigation by *Caetano et al.* from 2022 had previously examined COVID-19 in relation to new-onset T1D in children from March 2020 to March 2021, and compared to the years before [26]. Our findings corroborated the previously reported increase in T1D cases from that center. The two sites, Dona Estefânia Hospital in Lisbon and Centro Hospitalar Universitário do Algarve in Faro, were geographically distinct and both rather large. We could not identify any changes in procedures or patient uptake which could explain the increases.

Although viral infections have long been suggested as having a role in T1D development, studies concerning SARS-CoV-2 infection as a precipitating factor are currently pointing in different directions. Overall, our study is in line with recent large register studies from Scotland [12] and Denmark [17] that did not find evidence of a link between PCR confirmed SARS-CoV-2 and incidence of T1D. The Danish child study is interesting in this regard, as Denmark had one of the highest test frequencies in the world [17]. Other large studies, e.g. from the U.S. [11, 27], have, however, observed a possible association between the COVID-19 pandemic and diabetes development. A German register study among children and adolescents reported a peaking T1D incidence about 3 months after the peak in COVID-19 cases, which could be due to direct viral effects on ß-cell function. Indirect factors, possibly related to control measures and thereby altered or delayed exposures to other infections or lifestyle changes, were however considered more likely [28].

Though it did not reach statistical significance, more T1D cases were seen in our study during 2020 and 2021, compared to the two previous years. This would, however, not necessarily be indicative of an association with SARS-CoV-2 infection per se, as child T1D rates have been gradually increasing for many years, even before the pandemic [29]. Thus, a publication based on data from the SWEET register concluded that the slope of rise in pediatric T1D cases had remained unchanged during the pandemic [15]. In the Danish study investigating the entire population below 30 years, an increase in T1D cases was noted in April-June 2021 compared to April-June 2015–2019, but based on SARS-CoV-2 test results this was not attributed to the virus [18]. A study from Japan reported an increasing trend in pediatric T1D cases over the past 23 years – a trend unchanged by the COVID-19 pandemic [30].

One reason for the conflicting results may be due to the various study designs applied. For instance, the U.S. studies used data derived from health care claim databases. In contrast, the two studies from Denmark [17, 18] and the one from Scotland [12] were based on national health registries. Our present investigation from Portugal represents yet another type, compiling clinical data from individual hospital units to make an overall estimate.

Importantly, the COVID-19 pandemic affected countries in various ways, with different lock-down schemes being implemented. In Portugal, very strict control measures were implemented, with full lock-downs occurring in both March 2020 and January 2021. Apart from COVID-19 cases, only the most essential in-person medical appointments were allowed, with teleconsultations instead being encouraged. This likely resulted in a high proportion of 34% of people reporting unmet health care needs during the early pandemic, a proportion that was significantly higher than in many other European countries [31]. Naturally, these control measures also affected the transmission of other viral infections that may in some instances trigger T1D development. This could affect comparisons between populations. Clear changes in the seasonality of T1D diagnosis was observed elsewhere, with more cases recorded during the summer of 2020 compared to previous years [15]. In our data from Portugal, we confirmed the previous observed pattern of more T1D cases during the colder winter months, with most cases being detected in January and February. Others have noted how the mutating strains of SARS-CoV-2 and behavioral changes such as social distancing and increased hygienic practices complicate the interpretation of results in relation to T1D [30]. Finally, there may also be genetic differences in COVID-19 susceptibility [30].

Interestingly, the authors from the previous Portuguese study on T1D and COVID-19 had identified a higher proportion of severe DKA and intensive care unit admissions during the first year of the pandemic [26]. In Germany, an increase in DKA during the early pandemic was reported among children with new-onset T1D, which lasted even after the number of COVID-19 cases had dropped. Here, it was concluded that concern of infection had led to a prolonged avoidance of necessary health care, resulting in more DKA cases [32]. In Finland, more cases of severe DKA were observed during the pandemic, a finding considered related to changes in parental behavior and health care accessibility [33]. A similar finding of an increased rate of severe DKA cases was done in Poland [34]. We did not have data on DKA severity, but we did not find a higher overall DKA incidence during the epidemic, nor did previous COVID-19 affect the risk of DKA in the multivariate analysis. However, our DKA analysis may have been limited by low numbers and only from two hospitals. In our data, we noted the lowest monthly rate of new-onset T1D in March 2020 (only two cases), supporting the fact that T1D diagnoses were delayed at that time. In the multivariate analysis, we observed higher odds of DKA in young children, which may be due to early diabetes symptoms being harder to recognize here. A family history of T1D tended to reduce the risk of DKA, which could be due to better parental awareness of diabetes and hence earlier detection [35].

Noteworthy, BCG vaccination was previously until 2016 part of the standard vaccination programme in Portugal. In our study, 81% of the patients were BCG vaccinated. It has been suggested how BCG can ameliorate the severity of viral respiratory infections [36], likely through *non-specific* beneficial effects on the innate immune system [37]. In the U.S., a study among adult T1D patients showed a protective effect of multiple BCG vaccinations on SARS-CoV-2 infection [38]. This should be considered when comparing to settings where BCG vaccination has not been routinely applied, and it would be interesting to compare T1D incidence in more countries with and without obligatory BCG vaccination.

Also of note, the proportion of GAD positive T1D patients tended to increase during the pandemic (after the first 1st of March, 2020). While the change was quite small in percentage, i.e. 86% vs. 79%, it could be hypothesized that this may have been due to changes in viral triggers for autoimmunity. A similar trend was, however, not observed for IA-2 antibodies.

Finally, others have observed how COVID-19 may cause dyslipidaemia, possibly both through direct inflammatory pathways and also indirectly through behavioral changes [39]. Though we observed a slight increase in T1D patients with elevated cholesterol and LDL levels during the pandemic, this did not reach statistical significance. The predominantly young age of our participants, however, needs to be taken into account.

**Table 2:** Risk factor analysis for diabetes ketoacidosis at first presentation

	Univariate		Multivariate *N* = 155	
	OR (CI)	*P*	OR (CI)	*P*
**Age (**years**)**^1^	0.98 (0.95-1.00)	0.05	0.97 (0.95-1.00)	0.04
**Gender**				
Female Male	1.00 (ref) 1.09 (0.57–2.11)	- 0.79	1.00 (ref) 1.10 (0.55–2.20)	- 0.78
**T1D family disposition**				
No Yes	1.00 (ref) 0.57 (0.26–1.27)	- 0.17	1.00 (ref) 0.52 (0.23–1.17)	- 0.11
**HbA1c**				
<=12.0% (ref) > 12.0%	1.00 (ref) 1.28 (0.68–2.42)	- 0.44	1.00 (ref) 1.37 (0.71–2.65)	- 0.36
**Prior COVID-19 infection**				
No Yes	1.00 (ref) 1.27 (0.41–3.96)	- 0.68	1.00 (ref) 1.32 (0.41–4.27)	- 0.65

^1^ Included as a continuous variable

*Note* For abbreviations used, OR denotes odds ratio, CI 95% confidence interval, T1D Type 1 diabetes, HbA1c glycated hemoglobin, COVID-19 coronavirus disease 2019

### Implications

Our investigation contributes to the ongoing debate on whether T1D development can be triggered by the SARS-CoV-2 infection. Essentially, it calls for further studies, both in local and more global contexts, involving multicenter setups to increase the number of participants. The severity of COVID-19 and T1D at the time of diagnosis should also be examined further in clinical studies.

Though we did not find a significant correlation between COVID-19 and new onset T1D, we cannot exclude that it does in fact exist, as smaller trends may not have been captured by our analysis. For instance, in the Canadian cohort study it was concluded that COVID-19 may have contributed to a 3–5% excess burden of diabetes at the population level [16]. That study mainly focused on non-insulin-dependent diabetes, as there were few cases of insulin-dependent diabetes (i.e. T1D).

While a considerable number of studies have investigated the association between T1D incidence and the COVID-19 epidemic, both in adults and in children, investigations concerning the course of new-onset T1D among SARS-CoV-2 infected patients are sparse. In our study, we report on fasting C-peptide levels at the time of diagnosis, but not serial measurements over time. Future studies should take this into consideration, as the pace of loss of ß-cell function in T1D has important clinical implications.

## Conclusion

We could not establish a significant temporal association between the COVID-19 pandemic and T1D incidence, nor in terms of changes in key metabolic parameters such as glucose, HbA1c and fasting C-peptide at the time of diagnosis. Frequency of DKA at the time of T1D diagnosis was not affected either. Any strong association between SARS-CoV-2 and T1D was therefore not supported.

## Data Availability

The data can be made available by contacting the corresponding author upon reasonable request, provided approval by the study senior author and participating hospital units.

## Abbreviations

ACE-2: Angiotensin-converting enzyme 2
BCG: Bacillus Calmette-Guérin
COVID-19: Coronavirus disease 2019
GAD: Anti-glutamic acid decarboxylase
DKA: Diabetes ketoacidosis
HbA1c: Glycated hemoglobin
SARS-CoV-2: Severe acute respiratory syndrome coronavirus 2
T1D: Type 1 diabetes
